# MRI Features of the Vomeronasal Organ in Dogs (*Canis Familiaris*)

**DOI:** 10.3389/fvets.2020.00159

**Published:** 2020-03-24

**Authors:** Michał Dzięcioł, Przemysław Podgórski, Ewa Stańczyk, Antoni Szumny, Martyna Woszczyło, Barbara Pieczewska, Wojciech Niżański, Józef Nicpoń, Marcin Adam Wrzosek

**Affiliations:** ^1^Department of Reproduction and Clinic of Farm Animals, Faculty of Veterinary Medicine, Wrocław University of Environmental and Life Sciences, Wrocław, Poland; ^2^Department of General Radiology, Interventional Radiology and Neuroradiology Wroclaw, Wrocław Medical University, Wrocław, Poland; ^3^Center of Experimental Diagnostics and Innovative Biomedical Technologies, Faculty of Veterinary Medicine, Wrocław University of Environmental and Life Sciences, Wrocław, Poland; ^4^Department of Chemistry, Wroclaw University of Environmental and Life Sciences, Wrocław, Poland; ^5^Department of Internal Diseases With Clinic for Horses, Dogs and Cats, Faculty of Veterinary Medicine, Wrocław University of Environmental and Life Sciences, Wrocław, Poland

**Keywords:** MRI, VNO, Jacobson's organ, dogs, semiochemical communication

## Abstract

According to current knowledge, the vomeronasal organ (VNO, Jacobson's organ) is the structure responsible for semiochemical signal detection. In dogs and other mammals, it is located close to the vomer and palatine processes of the incisive and maxillary bones. Although there are reports describing the anatomy and histology of this structure, there are limited available reports assessing this organ in live individuals and no direct visualization reports in dogs. The aim of this study was 2-fold: (1) preparation and optimization of a protocol for magnetic resonance imaging (MRI) examination of the VNO in a cadaver study with precise visualization and localization, and (2) characterization of the physiological VNO image features in MRI of live dogs. The first part of the study was performed on 10 beagle cadavers, the second on 8 live beagle dogs. For the VNO visualization, a 1.5T MRI (Philips® Ingenia) scanner and 20-channel digital head-neck spine coil were used (Philips®, Holland). The cadaver study allowed confirmation of the organ's location by the topical application of an MRI contrast agent (gadolinium) via the external entrance of the VNO canal. Accurate delineation of the VNO was obtained using a high resolution submillimeter three-dimensional T1-fast field echo (FFE) 3D sequence. Imaging of the VNO in 8 living dogs allowed the description of the morphological MRI features and direct evaluation of its shape and size. The results obtained demonstrate the ability to visualize the VNO *in vivo* and to evaluate its structure in dogs.

## Introduction

Although the VNO (vomeronasal organ, Jacobson's organ) was first discovered by Frederik Ruysch in 1732 and later described in more detail by Ludwig Jacobson in 1813, the functioning of this organ, which is responsible for detection of semiochemical communication signals and in dogs probably also gustation, is still not fully understood (1–5). There are many reports (including the use of immunohistological methods) describing the histological structure of the VNO in dogs and other species; however, there are still no publications concerning the visualization of this structure in living animals (6–16). The need for VNO imaging arises from its postulated possible involvement in various behavioral pathologies, including aggression in companion animals (17). VNO has been demonstrated as a possible infectious entry pathway for herpeviridae (18). Also, given the role of the VNO in semiochemical communication in dogs in the context of social interactions, the health status of this organ could significantly influence the welfare of these animals (12). Therefore, precise and ante-mortem VNO imaging can potentially help in the diagnosis and recognition of these clinical problems.

Magnetic resonance imaging (MRI) is a technique allowing soft tissue visualization (19). Although physiological features of the head anatomy of dogs obtained by MRI examination are available, they do not include a description of VNO features and its location (20–23).

The aim of this study was 2-fold: (1) preparation and optimization of magnetic resonance imaging (MRI) examination protocol for the VNO with precise visualization and localization in a cadaver study, and (2) description and characterization of the VNO imaging features in MRI of live dogs.

## Materials and Methods

### Experimental Design

The study was conducted in the Diagnostic Centre for Experimental and Innovative Biomedical Technology, Faculty of Veterinary Medicine, Wrocław University of Environmental and Life Sciences, Poland and was reviewed and approved by the Local Ethical Committee for the Affairs of Experiments on Animals (Resolution No. 83/2015 of 17.06.2015). The study was divided into two parts: a cadaver study (*n* = 10) to obtain precise location of the VNO for imaging, and VNO imaging protocol optimization in healthy live dogs.

The cadavers (5 beagle dogs; 3 males and 3 females,) were animals euthanized for natural reasons not connected with our study. Ten examinations were performed on the cadavers: five with contrast medium applied topically and five without. The cadaver studies were performed on animals stored no longer than 24 h from euthanasia in a 4°C refrigerator; none of the cadavers was frozen. All animals were free from any pathology within the head and neck region.

The second part of the study was performed on clinically healthy living animals (*n* = 9) (eight beagles and one dolichocephalic dog) belonging to the local Experimental Kennel. All animals were adult dogs (range: 3–6 y.o.) of both genders (4 males and 4 females-beagles and one dolichocephalic male). None of the animals involved exhibited any pathologies within the head and neck. While gathering the history from the Kennel administrator and staff taking care of the animals, possible behavioral problems in dogs were excluded. Dogs with upper respiratory tract deformation (severe nasal septum or nasal cavity deformation) were not included to the study.

### Anesthesia

Live animals were anesthetized with the same protocol. Sedation was performed with medetomidine (Cepetor®, CP-Pharma, Germany) 0.1 ml/10 kg and butorphanol (Torbugersic®, Zoetis, Poland) 0.1 ml/10 kg administered intramuscularly. General anesthesia induction was performed with propofol (Scanofol® Norbrook, Northern Ireland) 1 mg/1 kg intravenously. All live dogs were intubated and inhalation anesthesia was applied with isoflurane. The vapor setting was 3–4% at induction with oxygen flow at 60 ml/kg/min; after 5 min, it was reduced to 1.5–3% for maintenance with oxygen flow at 20 ml/kg/min (24).

### The Cadaver Study

Visualization of the VNO was performed with a 1.5T MRI scanner (Philips Ingenia®) and a 20-channel digital head-neck spine coil (Philips®, Holland). For accurate delineation and imaging optimization of the VNO high-resolution, T2-weighted images (slice thickness 2 mm) were acquired in transverse, sagittal and dorsal planes) and a T1-FFE 3D sequence was used to obtain submillimeter resolution images (0.3/0.3/0.3, 0.2/0.4/0.2, and 0.3/0.5/0.3 mm for the transverse, sagittal and dorsal planes, respectively).

**Figure 1:**
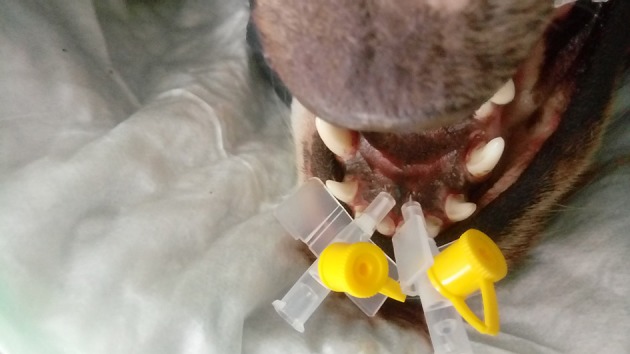
Two cannulas introduced into incisive papillae to instill the contrast medium in a cadaver model for precise localization of the VNO in the MR images.

In the first stage of the beagle cadaver study, 5 heads were scanned. The heads were placed in the head-neck spine coil in prone position. From these studies the precise location of the VNO was determined. The second stage of the cadaver study was performed to confirm the VNO location with topical contrast medium (gadolinium chloride, Dotarem®, Guerbet, France). The contrast agent was slowly infused through an intravenous cannula (size 24 G) placed in the external entrance of the nasopalatine duct, located in the palate, near the incisive papilla, behind the line of the upper teeth (Figure 1). Since the VNO canal opens into the nasopalatine duct, which indirectly connects the VNO duct to both the mouth (incisive papilla) and the nasal cavity, care was taken not to protrude the cannula inside the nasal cavity instead of VNO direction. In none of the cases was the cannula nor contract medium protruding into the nasal cavity.

### The Live Dog Study

Eight anesthetized adult beagle dogs were placed in ventral recumbency with their heads placed in the head-neck spine coil. Next, a high-resolution T-2 weighted and three-dimensional T1-FFE sequences, with pre- and post-contrast intravenous gadolinium chloride (Dotarem®, Guerbet, France) were acquired using the imaging parameters developed previously during the cadaver study.

To define the morphometric characteristics of the VNO, all MRI data obtained were analyzed with imaging software (EMS, Philips Medical Care®). The length, height and width of the VNO were measured.

Only the data obtained from the live beagles were used to describe the morphological features of the VNO (Table 1). The appearance of the VNO was described in T2-W and T1-W (pre- and post-contrast) sequences.

**Table 1:** Physiological features of the vomeronasal organ measured in live dogs: the length (L), width (W), and height (H) (mm).

		**LEFT**			**RIGHT**	
	**L (mm)**	**W (mm)**	**H (mm)**	**L (mm)**	**W (mm)**	**H (mm)**
1	22,1	1,6	3,5	21,6	2,5	3,4
2	25,2	1,0	1,1	26,1	1,1	1,2
3	26,1	2,6	2,3	25,3	2,0	2,2
4	27,8	2,0	2,3	28,5	2,1	2,0
5	22,7	2,0	3,1	23,1	2,1	2,7
6	25,1	2,0	3,3	26,1	1,9	3,5
7	24,1	1,8	2,8	22,2	2,0	1,8
8	26,9	2,6	2,6	27,8	2,7	2,6
Mean	25,0	1,9	2,6	25,0	2,0	2,4
St. Dev.	1,9	0,5	0,7	2,5	0,4	0,7

## Results

**Figure 2:**
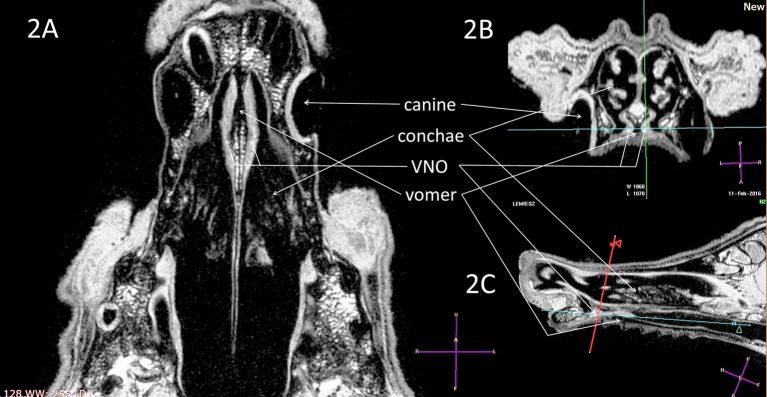
T1-weighted 3D images (naïve, without contrast), with visualization of the VNO in **(A)** dorsal, **(B)** transverse, and **(C)** sagittal projections. The green-blue cross shows a VNO location, corresponding to the blue-red lines crossing in the sagittal image. VNO, vomeronasal organ. The red, blue, and green lines correspond to levels of the transverse, dorsal, and sagittal projections, respectively.

**Figure 3:**
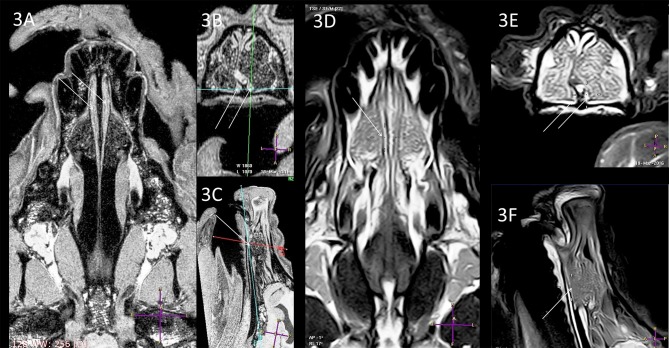
Comparison of the T1-w **(A–C)** and T2-w **(D–F)** images of the same VNO. Arrows and crossing lines show the locations of the VNOs. The VNO is T1-w isointense and T2-w hyperintense to the subcutaneous tissue with very good demarcation from the vomer bone in the T1-w images, whereas this is less detailed in the T2-w sequences. There is lateral deformation of the nasal septum **(B,E)** without influence on the VNO shape (**A**, dorsal view).

**Figure 4:**
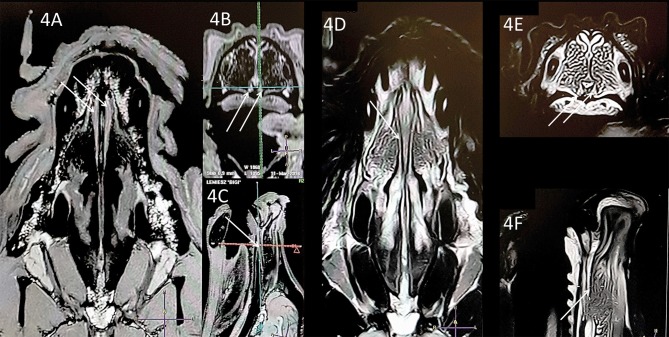
Comparison of the T1-w **(A–C)** and T2-w **(D–F)** images of the same VNO (white arrows). The nasal septum is straight (transverse projection, T1-w; 4B, T2-w; 4E) but this does not influence the size and shape of the VNO in comparison to the case in Figure 3.

**Figure 5:**
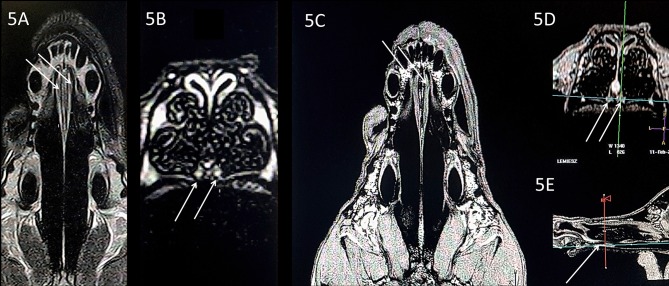
Comparison of the T1-w **(A,B)** and T1-w with contrast **(C–E)**, in a dolichocephalic dog. The elongated nasal cavity does not affect VNO visualization in comparison to the mesocephalic dogs (**Figures 2–4**). Uniform contrast enhancement is visible in the VNO, allowing for better delineation of the organ margins.

**Figure 6:**
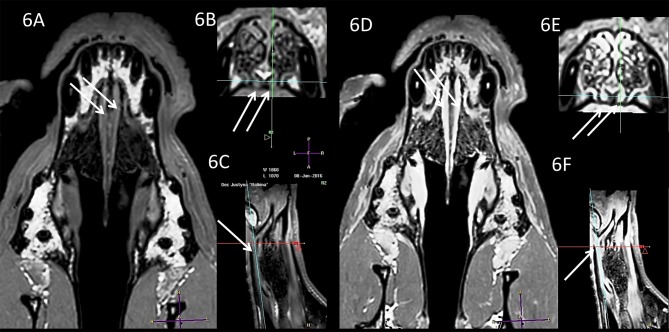
Comparison of the T1-w (**A**, dorsal; **B**, transverse; **C**, sagittal) and T1-w with contrast **(D–F)** in a mesocephalic dog. VNO (white arrows).

**Figure 7:**
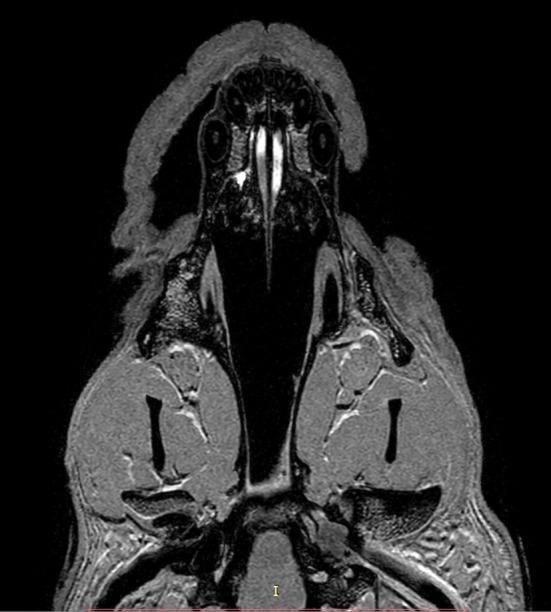
Visualization of the VNO (cadaver study) in a dorsal T1-w image after contrast material has been locally applied through the canal.

Accurate localization, identification and delineation of the VNO were obtained using high-resolution T2-W (slice thickness 2 mm), submillimeter sequences and three-dimensional T1-FFE sequences (0.3/0.4/0.3 mm) in both cadavers and live animals (Figures 2–**6**). The use of contrast medium introduced through the canal into the VNO in 5 cadavers improved the organ image quality and was used to confirm its location and identification from previously obtained non-contrast studies (Figure 7).

In all dogs the shape of the VNO was similar. In one case, a mildly-curved nasal septum was found (Figure 3). Despite the presence of this deformation, the VNO was not changed, therefore this anatomical anomaly did not influence VNO assessment and measurement (compare Figures 3 and 4: curved septum and straight septum).

Subjective analysis revealed that the best VNO assessment was obtained with dorsal projection (Figures 2A, 3A,D, 4A,D, 5A,C, 6A,D), because due to its “elongated water drop” shape and bilateral location on the vomer bone, this plane gave the largest organ view and the best possibility of comparing the two organs. There were no specific differences in the radiological features of VNO between the cadaver and live dogs studies. The use of topical contrast medium in the cadaver study enhanced the VNO and helped in its identification and localization. Intravenous administration of contrast medium in the live dogs caused homogenous enhancement of the whole organ with clear delineation of its margins (Figures 5, 6). The character of the VNO signal in the T2-W sequence was isointense to the conchal mucous tissue, hyperintense to subcutaneous tissue and hypointense to maxillary bone marrow (Figures 3D–F, 4D–F). Its appearance in the T1-FFE 3D sequence was isointense to the gray matter of the brain, subdermal connective tissue and hard palate tissue, hyperintense to cortical bone and slightly hypointense to the maxillary bone marrow (Figures 4, 5). After intravenous contrast administration, the VNO showed intense contrast enhancement similar to the conchal mucosa (Figures 6A–C pre-contrast vs. Figures 6D–F post-contrast). Measurement of the VNO (excluding the dolichocephalic dog) revealed mean lengths of 25,0 mm (left), 25,0 mm (right); heights 2,6 mm (left), 2,4 mm (right); and widths 1,9 mm (left), 2,0 mm (right) (Table 1).

## Discussion

The vomeronasal organ in dogs is located near the arms of the vomer bone, just above the roof of the mouth. The entrance leading through the nasopalatine duct into the VNO could be found in dogs just behind the line of the upper incisors. The VNO is a tubular, C-shaped organ split into a pair, separated by the nasal septum. The internal lumen is lined with a pseudostratified epithelium, containing receptor, supporting and basal cells (13–15).

The VNO was first described in the nineteenth century and is well-known to anatomists and neurophysiologists (13–15, 25). However, it is still poorly described and illustrated in anatomical atlases, as well as in advanced imaging textbooks with respect to its CT (computer tomography) or magnetic resonance imaging (MRI) features (23). In most publications, its description focuses on the vomer as a bone structure (23) rather than a characterization of the organ itself. Also, reports describing visualization of pathological lesions located in this region usually do not include information about the VNO (20, 21, 26). This could be partially explained by insufficient information about the function of the VNO and lack of possibilities for its imaging. Nowadays, the increasing interest in VNO pathologies, which have been suspected in animals with behavioral disturbances, together with development of advanced imaging techniques such as MRI, provide the possibility of developing this interesting branch of veterinary medicine.

Visualization of the VNO and better recognition of its physiological features support the development of diagnostic approaches for recognition of pathology involving this organ. The reports of Asproni et al. (17, 27) present evidence of degenerative and inflammatory processes concerning VNO structures in cats and pigs. These reports clearly demonstrated that the VNO, similar to other organs, may be involved in pathological processes. Moreover, the above-mentioned authors suggested that there could be a connection between identified degenerative lesions and observed *in vivo* changes in behavior, expressed as deficits in social skills. Asproni et al. (17) reported a statistically significant correlation between inflammation of the VNSE (vomeronasal sensory epithelium) and intraspecific aggression.

At present there are few reports describing pathological processes involving the VNO. In those available, however, lesions were detected during histopathological examination, and thus were done *post mortem*. Intravital imaging of VNO features allows for comparison with potential pathological changes and further diagnoses of behavioral disturbances.

Magnetic resonance imaging is a technique that allows for precise soft tissue assessment; therefore, it appears to be the most suitable imaging technique for VNO visualization (28). The signal intensity of the VNO obtained in this study in the 3D T1-W sequence was similar to that of brain gray matter, subdermal connective tissue and hard palate tissue (Figures 2–7). Due to the organ's small size (mean width 1.9 mm and height 2.5 mm, Table 1), a submillimeter study was needed, acquired as a T1-weighted sequence. In order to obtain sufficient imaging quality from the VNO, we decided to perform a T1-W 3-dimensional analysis with a slice of 0.3/0.3/0.3 mm. This assured proper demarcation of its borders and adequate detail recognition for this organ size.

The signal obtained in the T2-W sequence corresponded to soft tissue, such as mucosal membrane of the conchae. Due to the technical limitations of the T2-W sequence, this part of the study was limited to 2 mm slice thickness as thinner T2-W slices resulted in a poor quality images that could not have been used for a proper VNO assessment. Due to the small size of the VNO, only one slice was useful in the dorsal and sagittal projections of the T2-W sequence, and multiple T2-W slices were only useful in the transverse plane.

Limitations of the study included the use of 1.5 Tesla scanner; 3T and higher strength of the magnet field would have provided better imaging detail. With 1.5T accuracy, a T1-W 3D sequence was found to be adequate for VNO imaging and the T2-W sequence was also adequate when a slice was placed exactly through the middle of the VNO in the dorsal and sagittal planes. We did not use other imaging sequences (e.g., FLAIR) as those were characterized by very poor tissue detail, and although this organ was visible, the examination was not repeatable. Another limitation was the lack of comparison of the VNO features in dogs of different ages. This might play an important role in the diagnosis of geriatric behavioral disturbances that are observed in senile dogs. Brachycephalic breeds were not involved because physiological VNO assessment could not have been fulfilled in those breeds, and this was the goal of the study. VNO in brachycephalic breed dogs could be massively deformed, and become unrecognizable in the MRI. Although one dolichocephalic dog was included in our study (Figure 5), the elongated nasal cavity did not affect VNO visualization in comparison to the mesocephalic dogs (Figures 2–4). Thus, the presented protocol could be probably sufficient for imaging VNO in these breeds. However, this needs to be verified in further studies including a greater number of dolichocephalic dogs.

This study supported visualization and optimization of a VNO imaging protocol in healthy mesocephalic dogs using MRI, with a description of the morphological VNO features in T1-W and T2-W pulse sequences. The results obtained provide a basis for the development of further VNO *in viv*o diagnostics and detection of pathology in animals with behavioral problems. High resolution imaging of the VNO can also allow further evaluation of its function using the VNO semiochemical stimulation combined with brain fMRI technique (functional magnetic resonance imaging). The potential reactivity estimation of this part of the nervous system, in the context of semiochemical communication in dogs, will be investigated in our further studies. This preliminary study allows the creation of a new evaluation platform for this structure and could provide new data expanding our ability to understand the exact function of the VNO. A recently described protocol of fMRI imaging performed on awake, unrestrained dogs, makes this kind of evaluation possible and desirable (22, 29–31). In addition, further studies involving higher resolution MRI in live animals in the context of breed, age, gender, reproductive status and behavior disorders seem to be indicated.

In conclusion, the MRI technique is sufficient for visualization of the VNO *post mortem* and in live animals with submillimeter 3D T1-W sequences. Visualization and radiological assessment of the VNO could be included in the usual protocol of head organ examination. Careful attention should be paid to this structure in the context of pathological changes. Also, it is necessary to conduct further studies with larger numbers of animals to provide more precise information about the possible relationships between the structure of the VNO and breed, age, gender and skull type.

## Data Availability Statement

All datasets generated for this study are included in the article/supplementary material.

## Author Contributions

MD conceived the original idea. MD, MAW, and AS designed and performed the experiments. MW, PP, and ES carried out the experiment. MW and BP contributed to sample preparation. WN and JN helped to supervise the project. MAW, WN, and JN collection and analyzing data. MD and MAW took the lead in writing the manuscript.

### Conflict of Interest

The authors declare that the research was conducted in the absence of any commercial or financial relationships that could be construed as a potential conflict of interest.
